# Meta-analysis of studies comparing conservative treatment with antibiotics and appendectomy for acute appendicitis in the adult

**DOI:** 10.1186/s12893-019-0578-5

**Published:** 2019-08-14

**Authors:** Zhengyang Yang, Feng Sun, Shichao Ai, Jiafeng Wang, Wenxian Guan, Song Liu

**Affiliations:** Department of General Surgery, Drum Tower Hospital, Medical School of Nanjing University, 321 Zhongshan RD, Nanjing, 210008 China

**Keywords:** Meta-analysis, Adult acute appendicitis, Conservative treatment, Appendectomy

## Abstract

**Background:**

Appendectomy is considered the first treatment choice for appendicitis. However, controversy exists since conservative therapy is associated with fewer complications than appendectomy for patients with acute appendicitis (AA). This meta-analysis aimed to compare the outcomes between conservative therapy and appendectomy in the management of adult AA.

**Methods:**

A literature search was performed to screen eligible clinical studies. Subgroup analyses of the uncomplicated population, complicated population and mixed population of randomized clinical trials were subsequently performed. Clinical outcomes included the overall effective rate of treatment, complication rate, relapse rate (reoperation rate) and overall length of stay (LOS).

**Results:**

Eleven trials totalling 2751 patients (conservative = 1463, appendectomy = 1288) were analysed. Patients receiving conservative treatment had a lower overall effective rate (OR: 0.11 ~ 0.17) and complication rate (OR: 0.21 ~ 0.51). The conservative group had a higher reoperation rate (5.6, 95% CI: 3.1% ~ 10.2%) than the appendectomy group (OR: 9.58 ~ 14.29). Conservative treatment was associated with a shorter overall length of stay (0.47 day, 95% CI: 0.45 ~ 0.5 day) than appendectomy.

**Conclusions:**

For both uncomplicated and complicated adult AA, non-operative management with antibiotics was associated with significantly fewer complications and a shorter length of stay but a lower effective rate and higher relapse rate.

## Background

Acute appendicitis (AA) is probably the most common surgical emergency worldwide, and one in ten people will have AA during their lifetime [1]. Appendectomy has been the standard treatment for AA for more than a century. Although appendectomy is a routine surgical procedure with low mortality, it can be associated with postoperative morbidity [2].

Since Fitz et al. described the relationship between the appendix and pelvic abscess in 1886, which result in high mortality, appendectomy became the preferred treatment for AA [3, 4]. In the absence of antibiotics, appendectomy can reduce the risk of uncontrolled pelvic infection to save lives. Bailey et al. described the conservative management of appendicitis in 1930, including resting and fasting followed by delayed elective appendectomy [5]. Though appendectomy was the mainstay treatment, antibiotics were available. Coldrey E reported using antibiotic therapy to treat 471 AA in 1956 with low mortality (0.2%), and only 14.4% patients had recurrence [6]. Eriksson S reported no different efficacy between antibiotics and appendectomy in a randomized clinical trial (RCT) in 1995 [7]. In the past 10 years, conservative treatment has seemed to be safe and may represent an effective first-line treatment of AA, although with an unknown long-term risk of recurrence or other complications [8–10].

In consideration of the lifetime incidence of appendicitis, the choice of treatment may have the potential to impact many patients [11, 12]. The aim of the present meta-analysis was therefore to compare four outcomes in patients with AA, including uncomplicated and complicated populations managed with appendectomy or antibiotics. In addition, we performed subgroup analysis of all RCTs to evaluate the high-level evidence.

## Methods

### Search strategy

Clinical trials comparing conservative management with appendectomy as the primary treatment for AA in adults were eligible for inclusion. We searched clinical trials within Medline, Embase and the Cochrane Library (CDSR, CENTRAL, DARE). Regional databases such as CNKI, VIP, Wanfang and Unpublished or and the research database Clinicaltrials.gov were also included in our meta-analysis (1990.1.1–2017.07.31). The medical subject heading term was appendicitis with search terms appendiceal abscess, appendiceal phlegmon, appendiceal perforation, appendiceal gangrene and appendectomy, delayed operation, non-operation, conservat, antibiotic. For example, the search strategy in PubMed was as follows: ((((((((appendicitis [MeSH Terms]) OR appendiceal abscess [Title/Abstract]) OR appendiceal phlegmon [Title/Abstract]) OR appendiceal perforat [Title/Abstract]) OR appendiceal gangrene [Title/Abstract]) OR appendicular abscess [Title/Abstract]) OR appendicular phlegmon [Title/Abstract]) OR appendicular perforat [Title/Abstract]) OR appendicular gangrene [Title/Abstract]) AND ((appendectomy [Title/Abstract]) OR (appendicectomy [Title/Abstract]) OR (delay operation [Title/Abstract]) OR (delay surg [Title/Abstract]) OR (nonoperat [Title/Abstract]) OR (non-operat [Title/Abstract]) OR (nonsurg [Title/Abstract]) OR (non-surg [Title/Abstract]) OR (conservat [Title/Abstract]) OR (antibiotic [Title/Abstract]) OR (antiinfect [Title/Abstract]) OR (antiinfect [Title/Abstract])). Two authors (Shichao Ai and Jiafeng Wang) independently searched the databases, and three authors (Zhengyang Yang, Feng Sun and Song Liu) reviewed the extracted studies independently.

### Study selection criteria

**Fig. 1:**
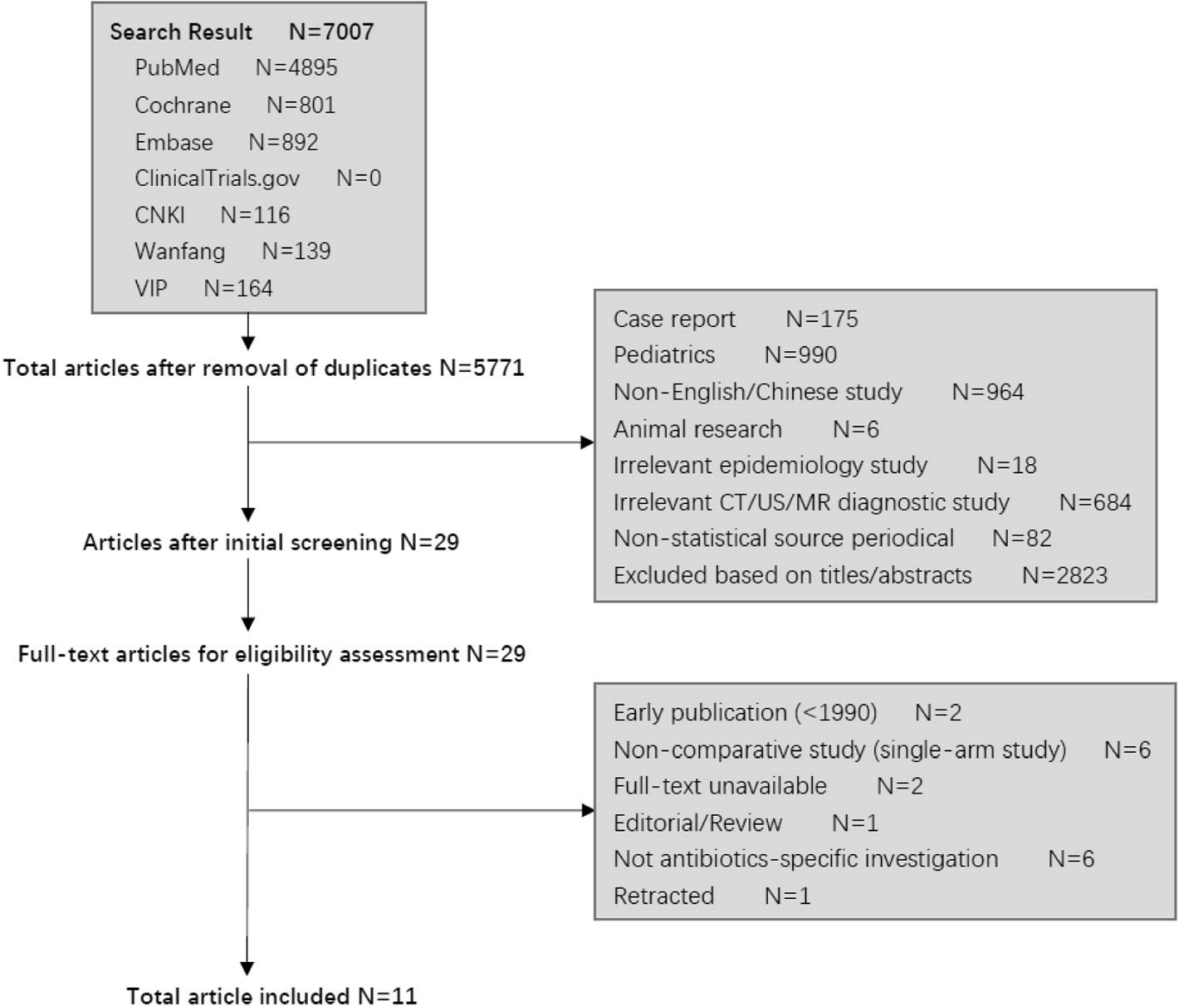
Flow chart of study selection

We included studies with all adults suspected or diagnosed with AA. Patients were divided into three subgroups: uncomplicated populations, complicated populations and RCT populations. All types of antibiotic, durations of antibiotic, and surgical technique (open and laparoscopic) were not exclusion criterions. Only Chinese- and English-language studies were eligible for inclusion. We excluded early publications (< 1990), case reports, editorials/reviews, paediatric studies, single-arm studies (non-comparative studies), irrelevant epidemiology studies, irrelevant CT/US/MR diagnostic studies, etc. (Fig. 1).

### Outcome measure

Three major outcomes were extracted: overall effective rate, recurrence of appendicitis and mortality. Minor outcomes included any antibiotic-related or surgery-related morbidity (surgical site infections, incisional hernias, abdominal, incisional pain, obstructive symptoms, abscesses, wound rupture, bladder dysfunction, diarrhoea, abdominal discomfort, etc.), length of hospital stay and length of sick leave. For conservative treatment, efficacy was defined as a definitive improvement in symptoms and without requiring an operation during the follow-up period. For appendectomy group, efficacy was defined as appendicitis confirmed by the operation or histological verification and resolution of clinical symptoms after the operation. Relapse rate (reoperation rate) in conservative group referred to patients that converted to surgical treatment while in appendectomy group referred to patients need a second operation.

### Statistical analysis

Statistical analysis was completed using RevMan 5.3 (The Nordic Cochrane Centre, The Cochrane Collaboration, Copenhagen, Denmark). We used the recommendations of The Cochrane Collaboration to obtain the meta-analysis results. The odds ratios (ORs) were assessed using the Cochran Q-test, assuming heterogeneity, with 95% confidence intervals (CIs) that did not include 1. The primary outcome measure was performed using the Mantel-Haenszel method. The effective rate of treatment, complication rates and reoperation rate are reported using ORs with 95% CIs. The weighted mean differences (WMDs) with the 95% CI and a random-effects model were used to assess length of stay.

### Evaluation of methodological quality

The methodological quality of the RCTs was evaluated by the Cochrane bias assessment tool in RevMan 5.3 with six criteria, including random sequence generation, allocation concealment, blinding of participants and personnel, blinding of outcome assessment, incomplete outcome data and selective reporting. Each study was determined to be at high or low risk of bias. The Newcastle-Ottawa scale (NOS) was used to evaluate the methodological quality of retrospective and prospective cohort studies. The NOS aspects of retrospective studies were patient selection, comparability and exposure, while those of prospective cohort studies were patient selection, comparability and outcome. Studies were deemed high quality if their aggregate score reached 5 or higher [13].

## Results

The search retrieved 4985 articles from the PubMed database, 801 articles from the Cochrane Library, 892 articles from the Embase database, 116 articles from the CNKI database, 164 articles from the VIP database, and 139 articles from the Wanfang database. The total number was 5771 articles after removal of duplicates. A total of 2823 articles were excluded based on titles/abstracts. The initial screening excluded 2919 studies, 175 because they were case reports, 990 because they were in paediatrics, 964 because they were not written in English or Chinese, 6 because they were animal research, 18 because they were irrelevant epidemiology studies, 684 because they were irrelevant CT/US/MR diagnostic studies, and 82 because they were non-statistical source periodicals. Twenty-nine articles were evaluated for full-text review. Two full texts were unavailable, while 2 early publications (< 1990), 6 single-arm studies, 1 review, 6 non-antibiotic-specific investigations, and 1 retracted article were eliminated. Finally, eleven studies [7, 14–23] including 2751 patients were included in our meta-analysis (Fig. 1).

The eleven studies were 5 RCTs, 3 retrospective studies and 3 prospective cohort studies. From the brief information and each trial’s methodology, we saw that all studies had two arms, conservative and appendectomy. Conservative management included antibiotic strategies and other conservative strategies. Two studies had a sample size of < 50 per group. There were differences in the choice of antibiotic strategy, whose details are summarized in Table 1. Six studies had other conservative strategies, such as waiting to see if the patient improved within 24 h and performing appendectomy if not. These trials included 2751 patients, 1463 treated with conservative treatment and 1288 treated with surgery (Table 1).

**Table 1:** Brief information of the included studies

First author	Eriksson S	Oliak D	Tingstedt B	Styrud J	Liu K	Hansson J	Turhan A	Vons C	Hansson J	Mentula P	Salminen P
Year	1995	2001	2002	2006	2007	2009	2009	2011	2012	2015	2015
Journal	Br J Surg	Dis *Colon rectum*	Eur J Surg	World J Surg	Am Surg	Br J Surg	Ulus Travma Acil Cerrahi Derg	Lancet	World J Surg	Ann Surg	JAMA
Region	Sweden	USA	Sweden	Sweden	USA	Sweden	Turkey	France	Sweden	Finland	Finland
Study type	Prospective controlled	Retrospective	Retrospective	Multicentre RCT	Retrospective	Multicentre RCT	Prospective controlled	Multicentre RCT	Prospective nonrandomized	Single centre RCT	Multicentre RCT
No. conservative group	20	88	50	128	19	202	107	120	442	30	257
No. surgery group	20	67	43	124	51	167	183	119	111	30	273
Patient type	Mixed	Periappendiceal abscess	Periappendiceal abscess	Mixed	Uncomplicated	Mixed	Mixed	Uncomplicated	Mixed	Periappendiceal abscess	Uncomplicated
Antibiotic strategy	(cefotaxime 2 g bid + tinidazole 0.8 g qd) iv 2d + (ofloxacin 0.2 g bid + tinidazole 0.5 g bid) po 8d	iv administration, N/A	(cephalosporin + metronidazole OR imipenem) iv + (cephalosporin + metronidazole) po	(cefotaxime 2 g bid + tinidazole 0.8 g qd) iv 2d + (ofloxacin 0.2 g bid + tinidazole 0.5 g bid) po 10d	iv 1-6d + po 7-14d	(cefotaxime 1 g bid + metronidazole 1.5 g qd) iv 1d + (ciprofloxacin 0.5 g bid + metronidazole 0.4 g tid) po 10d	(ampicillin 1 g qid + gentamicin 160 mg qd + metronidazole 0.5 g tid) iv 3d + N/A po 7d	amoxicillin/clavulanic acid (3 g/d if BW < 90 kg, 4 g/d if BW > 90 kg), iv OR po	(piperacillin/tazobactam 4 g tid) iv 1d + (ciprofloxacin 0.5 g + metronidazole 400 mg bid) po 9d	(cefuroxime 1.5 g tid + metronidazole 0.5 g qd) iv + (cephalexin 0.5 g tid + metronidazole 0.5 g tid) po 7d	(ertapenem 1 g qd) iv 3d + (levofloxacin 0.5 g qd + metronidazole 0.5 g tid) po 7d
Other conservative strategy	N/A	PCD selectively, if not improve in 72 h will receive appendectomy	PCD (18%, 9/50)	if not improve in 24 h will receive appendectomy	N/A	N/A	N/A	if not improve in 48 h will receive appendectomy	if not improve in 24–48 h will receive appendectomy	PCD if abscess> 3 cm	N/A
Conflict of Interests	Swedish Hoechst AB, Pfizer AB, Mutual Group Life Insurance Company ‘Forenade Liv’	N/A	N/A	Wallenius Corporation, Aventis Pharma	N/A	None	N/A	None	None	None	Merck, Roche

### Evaluation of methodological quality

All RCTs in our meta-analysis had different risks of bias. None of the studies blinded participants or personnel. Fortunately, none of our RCTs met more than half of the risk-of-bias criteria (Table 2). For the NOS results, the aggregate score of all retrospective studies (Table 3) and prospective cohort studies (Table 4) reached 5 points or higher. These results indicate the high methodological quality of studies included in our meta-analyses.

**Table 2:** Risk-of-bias summary of randomized clinical trials

Reference	Styrud J 2006	Hansson J 2009	Vons C 2011	Mentula P 2015	Salminen P 2015
Random sequence generation	+	+	+	+	+
Allocation concealment	+	+	+	+	+
Blinding of participants and personnel	–	–	–	–	–
Blinding of outcome assessment	–	+	+	+	+
Incomplete outcome data	–	+	+	+	/
Selective reporting	/	/	/	/	/
Other bias	/	/	/	/	/

+: low risk of bias

-: high risk of bias

/: unclear risk of bias

**Table 3:** Methodological quality criteria in retrospective studies

Reference	Oliak D 2001	Tingstedt B 2002	Liu K 2007
Patient selection			
Definition adequate	1	1	1
Representativeness	1	1	1
Selection of controls	0	0	0
Definition of controls	1	1	1
Comparability			
Most important factor	1	1	1
Other additional factor	0	0	0
Exposure			
Ascertainment of exposure	1	0	0
Same method of ascertainment for cases and controls	1	1	1
Non-response rate	0	0	0
Aggregate score	6	5	5

**Table 4:** Methodological quality criteria in prospective controlled studies

Reference	Eriksson S 1995	Turhan A 2009	Hansson J 2012
Patient selection			
Representativeness	1	1	1
Selection of the non-exposed cohort	1	1	1
Ascertainment of exposure	0	1	1
Demonstration that outcome of interest was not present at start of study	1	0	1
Comparability			
Most important factor	1	1	1
Other additional factor		0	0
Outcome			
Assessment	0	0	1
Follow-up long enough for outcomes to occur	0	1	1
Adequacy of follow-up	1	1	0
Aggregate score	5	6	7

### Outcomes

#### Effective rate of treatment

The overall effective rate of conservative treatment in adult appendicitis was 82.8 (95% CI: 77.2% ~ 88.2%). That in the uncomplicated population was 95.2% (95% CI: 84.4% ~ 98.4%), the complicated population was 83.4% (95% CI: 57.8% ~ 94.4%), and the RCT population was 74.1% (95% CI: 66.4% ~ 82.2%).

**Fig. 2:**
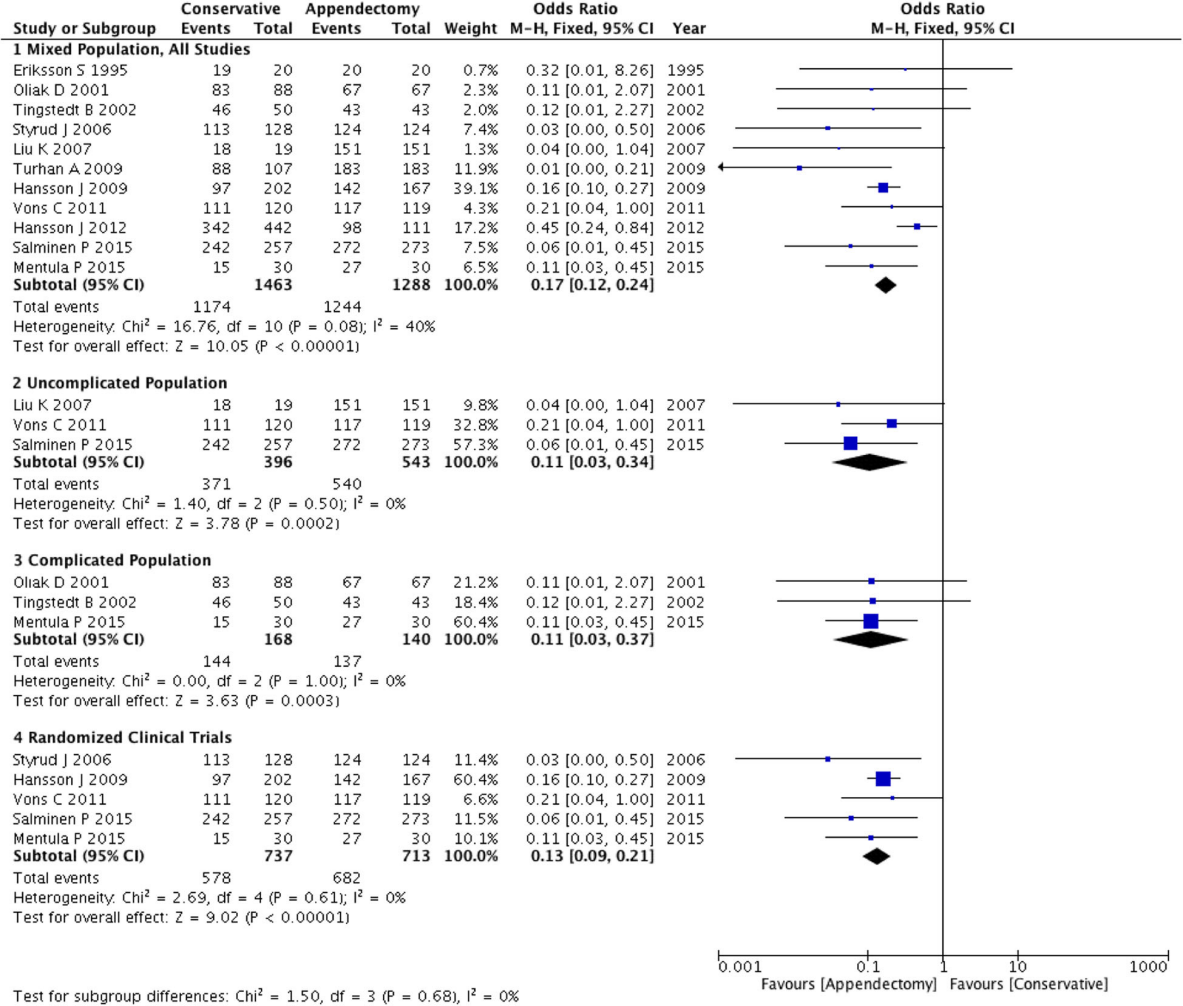
Forest plot showing the effective rate of both conservative and appendectomy treatments in the three subgroups: mixed population, uncomplicated population and complicated population

Meta-analysis of the effective rate showed a significant reduction in conservatively managed compared with appendectomized patients in all three subgroups (OR: 0.11 ~ 0.17). Test for subgroup differences: χ^2^ = 1.50, df = 3 (*P* = 0.68), I^2^ = 0% (Fig. 2).

#### Complication rate

**Fig. 3:**
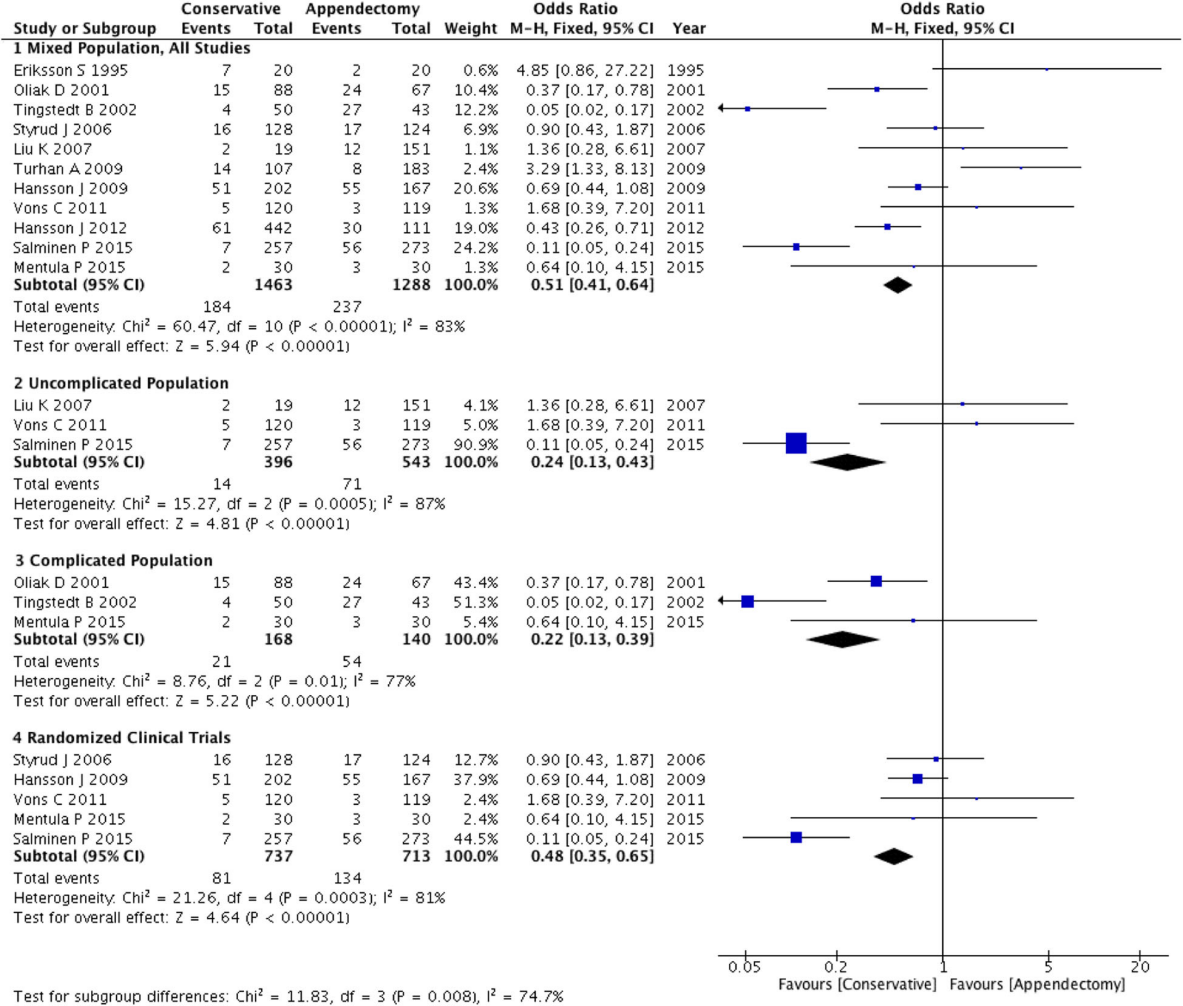
Forest plot showing the complication rate of both conservative and appendectomy treatments in the three subgroups: mixed population, uncomplicated population and complicated population

The complication rate of all conservative patients was 10.3% (95% CI: 8.5% ~ 12.6%). The results in the three subgroups were uncomplicated: 3.5, 95% CI: 1.9% ~ 6.1%; complicated: 12.1, 95% CI: 7.5% ~ 19.7%; and RCT: 10.0, 95% CI: 7.5% ~ 13.1%. The incidence of complications was all lower in these three subgroups than in the emergency appendectomy group (OR: 0.22~0.51). Test for subgroup differences: χ^2^ = 11.83, df = 3, (*P* = 0.008), I^2^ = 74.7% (Fig. 3).

#### Relapse rate (reoperation rate)

**Fig. 4:**
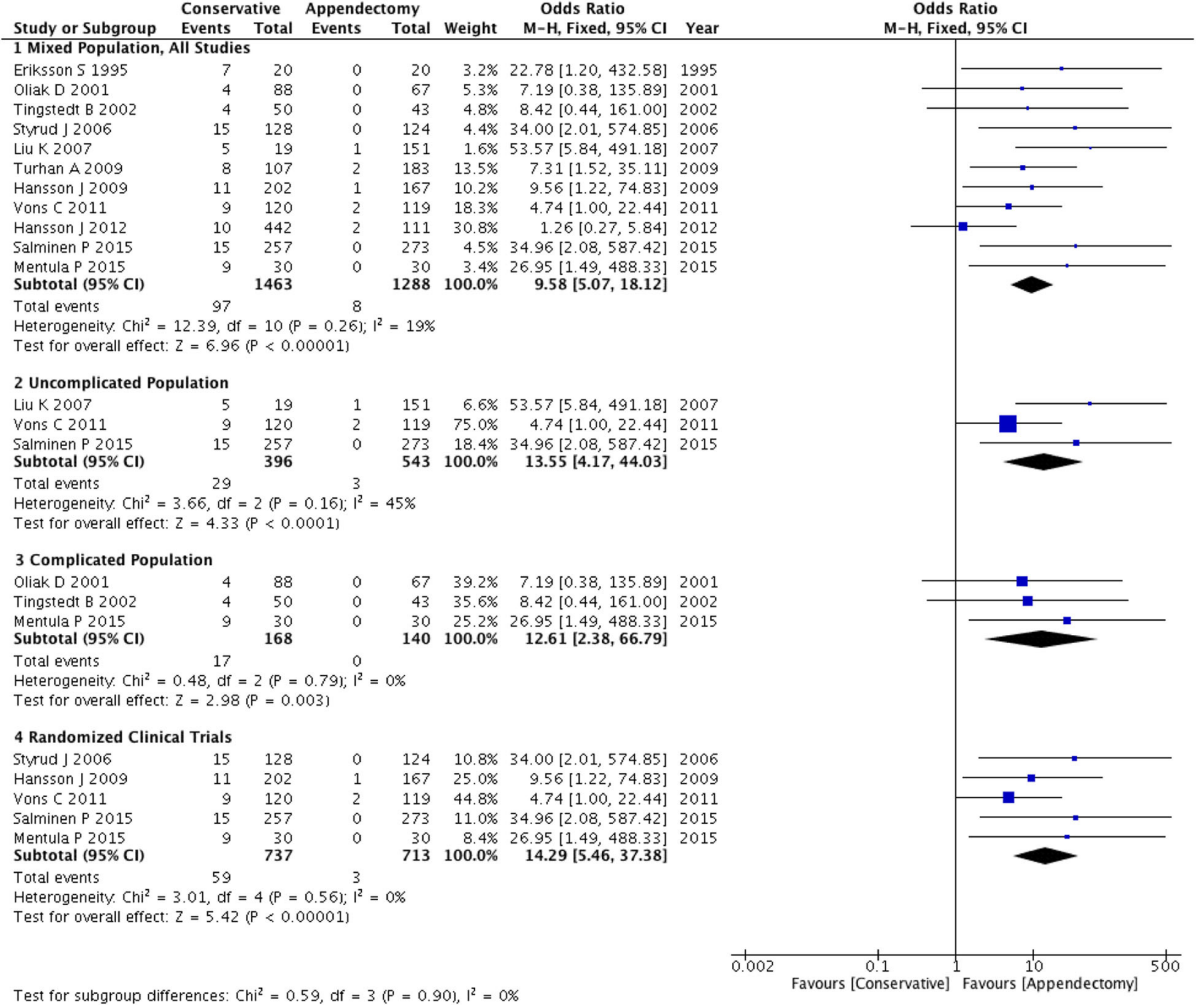
Forest plot showing the reoperation rate of both conservative and appendectomy treatments in the three subgroups: mixed population, uncomplicated population and complicated population

The reoperation rate of conservative treatment was 5.6% (95% CI: 3.1% ~ 10.2%). Two subgroups showed higher reoperation rates: RCT (5.7, 95% CI: 2.3% ~ 13.6%) and uncomplicated (7.0, 95% CI: 2.3% ~ 19.7%). The above evidence shows that the relapse rate after emergency appendectomy was lower than that after conservative treatment (OR: 9.58~14.29). Test for subgroup differences: χ^2^ = 0.59, df = 3, (*P* = 0.90), I^2^ = 0% (Fig. 4).

#### Length of stay (LOS)

**Fig. 5:**
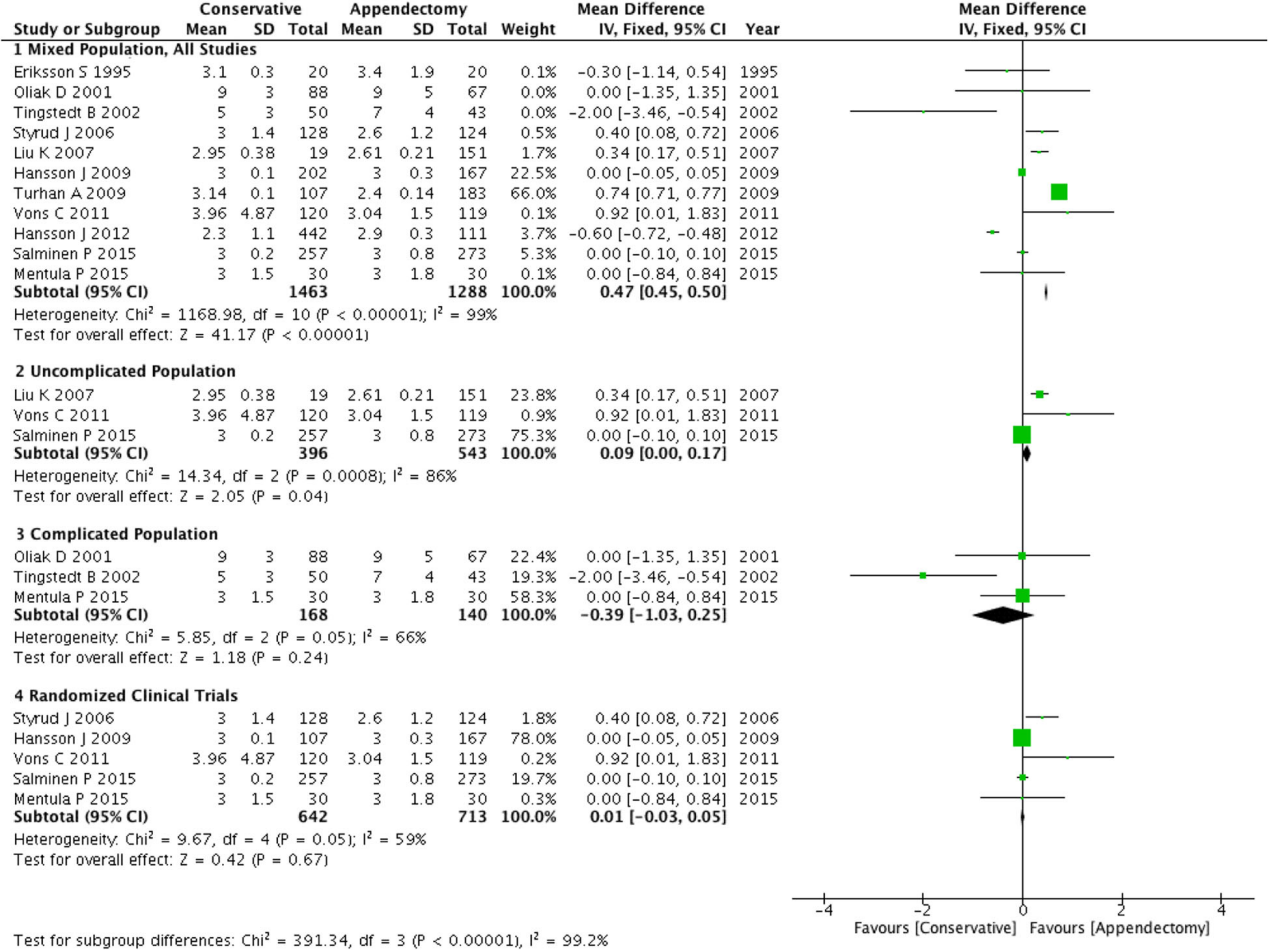
Forest plot showing the length of stay of both conservative and appendectomy treatments in the three subgroups: mixed population, uncomplicated population and complicated population

All studies reported the length of the primary hospital stay. Only one trial had a reduced sample size in the RCT subgroups [18]. The overall length of stay in the conservative group was 0.47 days (95% CI: 0.45 ~ 0.50 days) longer than that of the surgery group. In the RCT population, this difference was 0.01 days (95% CI: − 0.03 ~ 0.05 days), while in the uncomplicated population it was 0.09 days (95% CI: 0.00 ~ 0.17 days) and in the complicated population it was − 0.39 (95% CI: − 1.03 ~ 0.25 days). The forest plot of the comparison of length of stay also showed the difference between the two groups. Test for subgroup differences: χ^2^ = 391.34, df = 3, (*P* < 0.00001), I^2^ = 99.2% (Fig. 5).

## Discussion

Currently, emergent appendectomy is still the primary treatment choice for AA because of its low mortality and low rate of recrudescence and perforation. Our meta-analysis contained more than 2700 patients to compare the advantages and disadvantages between conservative treatment and appendectomy. Advantages of appendectomy include higher overall effective rates of treatment and lower reoperation rates. These advantages need to be considered along with higher complication rates and potentially longer hospital stay.

Randomized controlled trials are a means that divide the subjects randomly into different groups and apply different interventions accordingly. It is recognized as the gold standard for evaluating an intervention measure because it has the advantages of avoiding various biases, balancing the confounding factors and improving the effectiveness of the statistical tests. We added a subgroup of RCTs to make our data more convincing with less deviation.

A recent meta-analysis compared the efficacy of conservative and appendectomy therapy in uncomplicated AA [24]. They found treatment efficacy rate of 72.6 and 93.1% in two groups, respectively, which is comparable with our data (80.2% VS 96.6%). The difference might mainly originate from patient selection and definition of efficacy. Another recent meta-analysis reported the complication rate of 11.6 and 19.0% in conservative and appendectomy groups, which was consistent with our data (12.6% VS 18.4%) [25].

Conservative treatment can avoid emergency surgery, avoid the relatively high complication rate of emergency surgery, and give simple appendicitis patients a shorter hospitalization time, and it is often favoured by both doctors and patients [26]. Conservative treatment of antibiotics has been widely used in the clinic, and it has a higher resource utilization rate. Conservative treatment-related puncture and drainage technology has also been widely adopted in clinical practice [25]. Many studies have compared the efficacy of emergency appendectomy with it [27, 28]. Conservative treatment is easy to carry out in emergency and outpatient appendicitis, and the related drugs are easy to obtain. At present, there are few high-quality studies or large-sample cost-benefit analyses to evaluate the advantages and disadvantages of conservative treatment.

The efficacy of conservative treatment was a highly debated issue in different studies. [29–31]. In our meta-analysis, the definition of efficacy in conservative treatment mainly comes from the original literature, i.e., a definitive improvement in symptoms and without requiring an operation during the follow-up period. However, the duration of follow-up period varies from 60 days to 1 year among different studies. Some studies did not even define the duration of follow-up. This could become one of limitations of our meta-analysis. In addition, the general lifetime risk of 6.7–8.6% for appendicitis persists in conservative treatment group should also be considered [1]. We recommend to define the efficacy as success of initial treatment without a recurrence during the follow-up of 1 year, because this standard was commonly used currently [19, 22, 24, 25, 32].

As for international guidelines on the recommendation of conservative treatment for AA, EAES 2015 holds that appendectomy remains the gold standard in acutHe uncomplicated appendicitis, while it is difficult to draw firm conclusions regarding the treatment of complicated appendicitis [33]. SAGES 2010 is inclined to discuss the safety, efficacy and indication of endoscopic appendectomy, and it does not recommend conservative treatment of AA [34]. WSES 2016 tells us that antibiotic therapy can be successful in selected patients with uncomplicated appendicitis who wish to avoid surgery and accept the risk of recurrence (up to 38%). Meanwhile, non-operative management is a reasonable first-line treatment for appendicitis with phlegmon or abscess [35].

We are aware of the limitations of our study. First, the combined analysis of complicated and uncomplicated AA might result in publication bias due to inconsistent practices among different medical centres. Second, different antibiotic therapies in conservative treatment could become another potential bias. Third, in addition to antibiotic therapy, other conservative treatments (such as drainage) can affect the outcome but can hardly be evaluated in the comparison of conservative treatment with surgery. Fourth, a series of parameters including white blood cells, C-reactive protein, body mass index and severity of symptoms could affect the result of clinical treatment [36]. This aspect should but could not be included into current analysis because relevant data was not provided by the original literature. Fifth, the time from the diagnosis to the treatment was an important element that may affect the outcome of therapy, which however was not included into our study due to the lack of data in the original literature. Sixth, complications should be classified into different severity levels by Clavien-Dindo scoring system [37, 38], which unfortunately cannot be performed due to the lack of relevant data in the original literature.

## Conclusions

According to our meta-analysis, we can draw the follow recommendations. For adult patients with AA, conservative treatment has a high efficiency, although still slightly lower than that of appendectomy, but its incidence of complications is significantly lower than that of emergency surgery. Therefore, for patients who do not have a strong desire for emergency surgery or refuse emergency surgery, a conservative treatment that mainly entails anti-infection may be temporary. Nevertheless, it is necessary to emphasize the risk of recurrence and converting to operation in conservative treatment, and the rate of reoperation is higher than that of emergency operation. All the above opinions apply to patients with uncomplicated and complicated appendicitis.

## Data Availability

The datasets used and analysed during the current study are available from the corresponding author on reasonable request.

## Abbreviations

AA: Acute appendicitis
CI: Confidence interval
LOS: Length of stay
NOS: Newcastle-Ottawa Scale
OR: Odds ratio
RCT: Randomized clinical trial
WMD: Weighted mean difference
